# The successful management of a cardia hamartomatous inverted polyp via endoscopic submucosal dissection: a case report

**DOI:** 10.3389/fsurg.2025.1682095

**Published:** 2025-10-09

**Authors:** Xu Zhang, Hong-nian Pan, Xiu-zhong Wang, Mei Li, Jie Liu, Xiao-yan Liu

**Affiliations:** 1 Department of Gastroenterology, Lu'an Hospital of Anhui Medical University, Lu'an, Anhui, China; 2 Department of Gastroenterology, Lu'an People's Hospital of Anhui Province, Lu'an, Anhui, China; 3 Department of Pathology, Lu'an Hospital of Anhui Medical University, Lu'an, Anhui, China; 4 Department of Gastroenterology, The First Affiliated Hospital of USTC, Division of Life Sciences and Medicine, University of Science and Technology of China, Hefei, Anhui, China

**Keywords:** cardia hamartomatous inverted polyp, submucosal tumor, endoscopic submucosal dissection, gastric cancer, endoscopic treatment

## Abstract

**Background:**

A gastric hamartomatous inverted polyp (GHIP) is an uncommon submucosal neoplasm that is histopathologically defined by a submucosal inverted growth of cystically dilated hyperplastic gastric glands.

**Case presentation:**

A 74-year-old Chinese man presented with a submucosal tumor (SMT) in the cardia, identified through electronic gastroscopy. This report presents a case of cardia hamartomatous inverted polyp (CHIP), which represent a rare histological variant of gastric polyps that pose diagnostic challenges. The endoscopic examination revealed the presence of a submucosal tumor, and endoscopic ultrasonography indicated a heterogeneous tumor predominantly situated within the third (submucosal) layer. Immunohistochemistry outcomes indicated MUC5AC (+), MUC6 (+), Syn (+), Ki-67 (+, approximately 5%), Desmin (+), SMA (+), as well as MUC2 (−). To achieve *en bloc* resection for lesions >1.0 cm, endoscopic submucosal dissection (ESD) was performed. The pathological evaluation confirmed the diagnosis of CHIP. The patient was discharged without experiencing any complications.

**Conclusion:**

Therefore, the ESD approach may be particularly suitable for the management of SMT-type hamartomatous inverted polyps.

## Background

An atypical form of gastric polyp, which resembles a submucosal tumor, has been identified as a hamartomatous polyp (1). These polyps are composed of the gastric mucosa and a submucosal component characterized by the proliferation of pseudo-pyloric glands, cystic glands, and bundles of smooth muscle (2). Despite being classified as hamartomatous or ectopic, the histogenetic origins of these polyps remain unresolved. The preoperative diagnosis of gastric hamartomatous inverted polyp (GHIP) poses significant challenges due to its infrequency. In this report, we present a case of a hamartomatous inverted polyp located in the cardia, exhibiting characteristics of a submucosal tumor (SMT), which was successfully excised using the endoscopic submucosal dissection (ESD) technique without any complications.

## Case report

A 74-year-old male patient was admitted to Lu'an People's Hospital for the evaluation and management of a SMT located in the cardia, measuring over 1.0 cm in diameter, which had been identified through upper gastrointestinal endoscopy. The primary complaint of the patient was abdominal bloating persisting for nearly 3 years, and no other familial conditions were detected, including cancers. The patient had not undergone gastroscopy or abdominal surgery before. The patient had irregularly taken proton pump inhibitors (PPIs) in the past due to abdominal bloating. The patient's abdominal bloating had shown minimal to no improvement with intermittent PPIs use over the 3-year period, which was a contributing factor in the decision to proceed with a comprehensive endoscopic evaluation to rule out an underlying structural cause. The patient had a history of hypertension for 2 years, with irregular administration of nifedipine sustained-release tablets, and his blood pressure was well-controlled. The patient denied having any other diseases or a history of previous surgeries. The patient is a farmer and has no mental or psychological issues.

**Figure 1:**
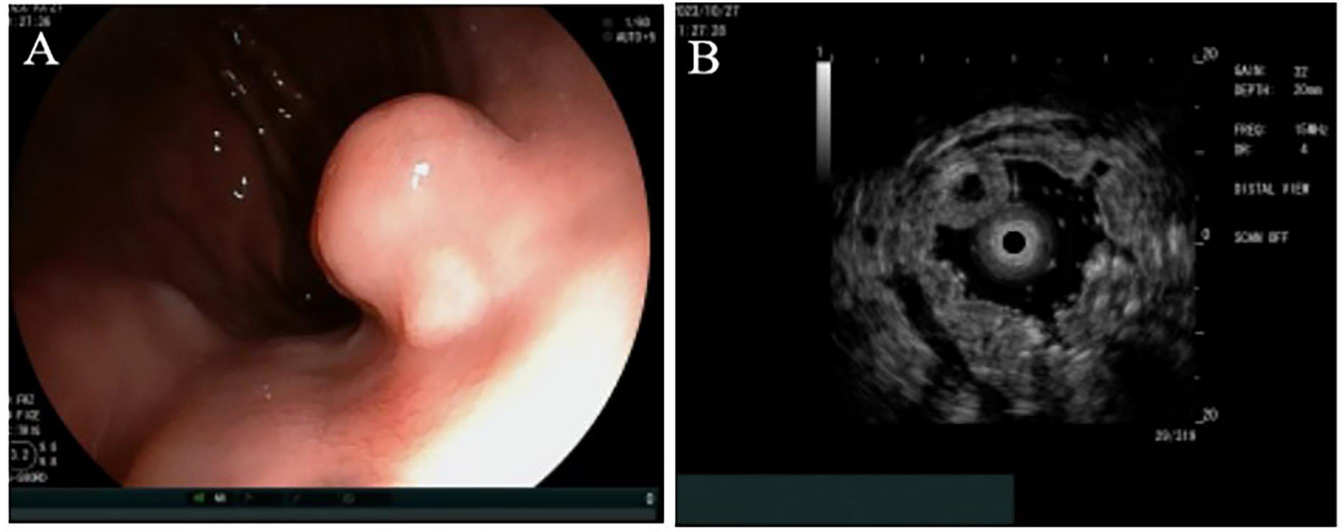
Endoscopic findings. **(A)** A hemispherical smooth submucosal mass was observed on white light endoscopy. **(B)** Endoscopic ultrasonography showed a hypoechoic mass with uniform internal echo originating from the third (submucosal) layer.

Upon examination, no pigmentation was observed in the oral cavity or on the lips. The patient's abdomen was flat and soft, without varicose veins on the abdominal wall. Vital signs such as respiratory rate and heart rate were within normal range, and there were no murmurs on auscultation of the heart and lungs. The physical examination and laboratory results upon admission did not reveal any abnormalities. Upper gastrointestinal endoscopy confirmed the presence of an SMT in the cardia, which exhibited no apparent erosion (see Figure 1A). Endoscopic ultrasonography indicated a heterogeneous tumor predominantly situated within the third (submucosal) layer (see Figure 1B). These observations raised the suspicion of a malignant neoplasm; however, a definitive diagnosis could not be established based solely on these findings. Consequently, it was deemed necessary to perform resection to obtain an accurate diagnosis and determine the most appropriate treatment. Given the challenges associated with conventional endoscopic mucosal resection (EMR) techniques, such as strip biopsy and EMR using a cap-fitted endoscope, we opted to utilize the ESD technique to achieve complete tumor resection in a single *en bloc*.

## ESD procedure

**Figure 2:**
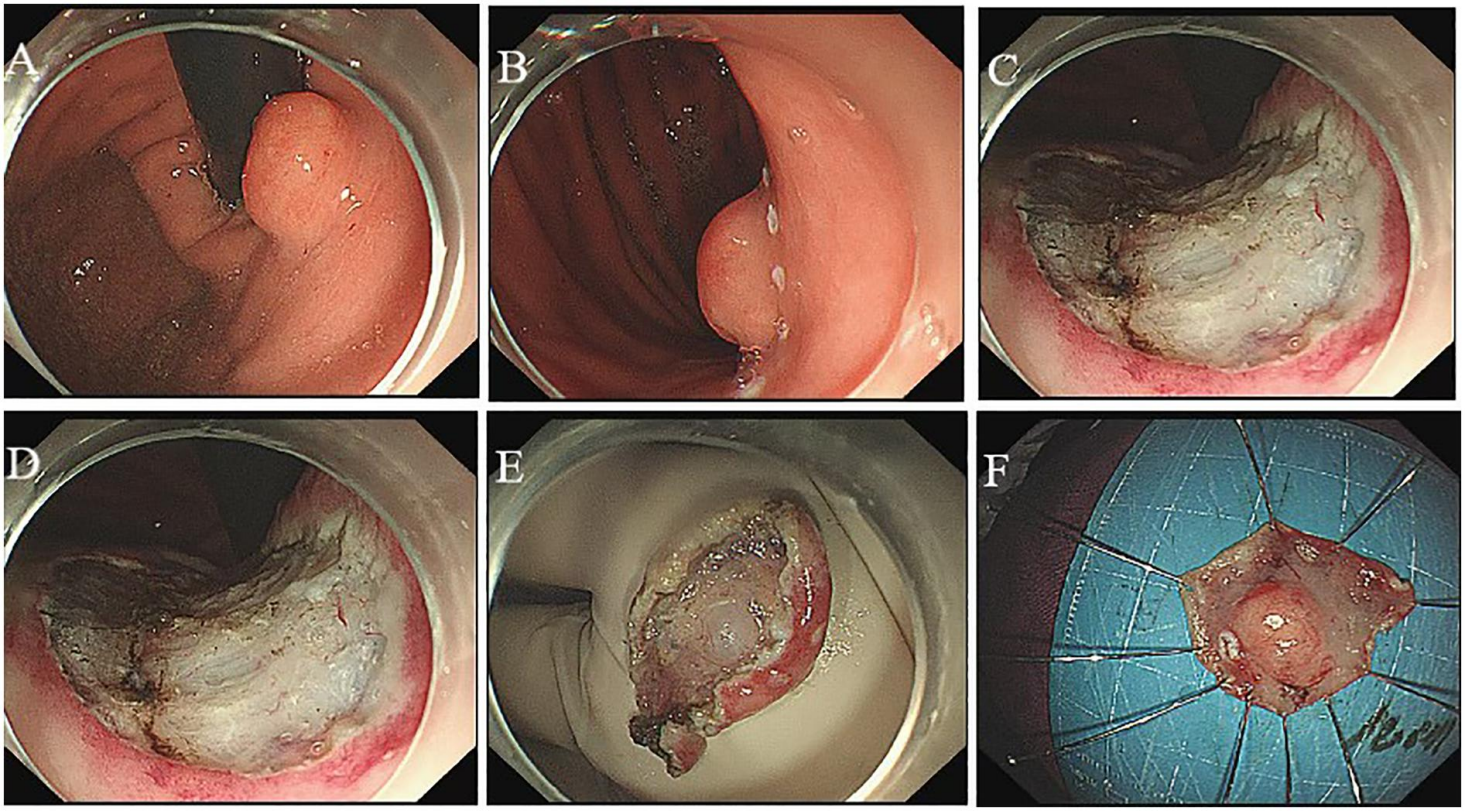
Endoscopic submucosal dissection. The flushing knife 2.0BT was used to cut along the margin to expose the submucosal white tumor **(A,B)**, the base of which was connected to the muscular layer and completely peeled along the margin of the tumor **(C–F)**.

The ESD procedure successfully resulted in the complete removal of the tumor, which measured 1.0 cm in diameter (see Figure 2). In details, The ESD procedure was performed under general anesthesia with the patient in the left lateral position. We used a standard single-channel gastroscope and a transparent cap attached to the tip. A mixed solution of indigo carmine, epinephrine (0.01%), and saline was injected into the submucosa around the lesion to create a cushion and lift the tumor. A circumferential mucosal incision was made around the lesion. Subsequent submucosal dissection was carefully performed using the same knife in Swift Coagulation mode, meticulously separating the tumor from the underlying muscularis propria layer to achieve *en bloc* resection. Hemostasis during the procedure was achieved using the knife in soft coagulation mode or with hemostatic forceps as needed. The resected specimen, measuring 1.0 cm × 1.0 cm, was retrieved with a Roth net and pinned flat on a foam board for formalin fixation and pathological assessment.

## Histological examination

**Figure 3:**
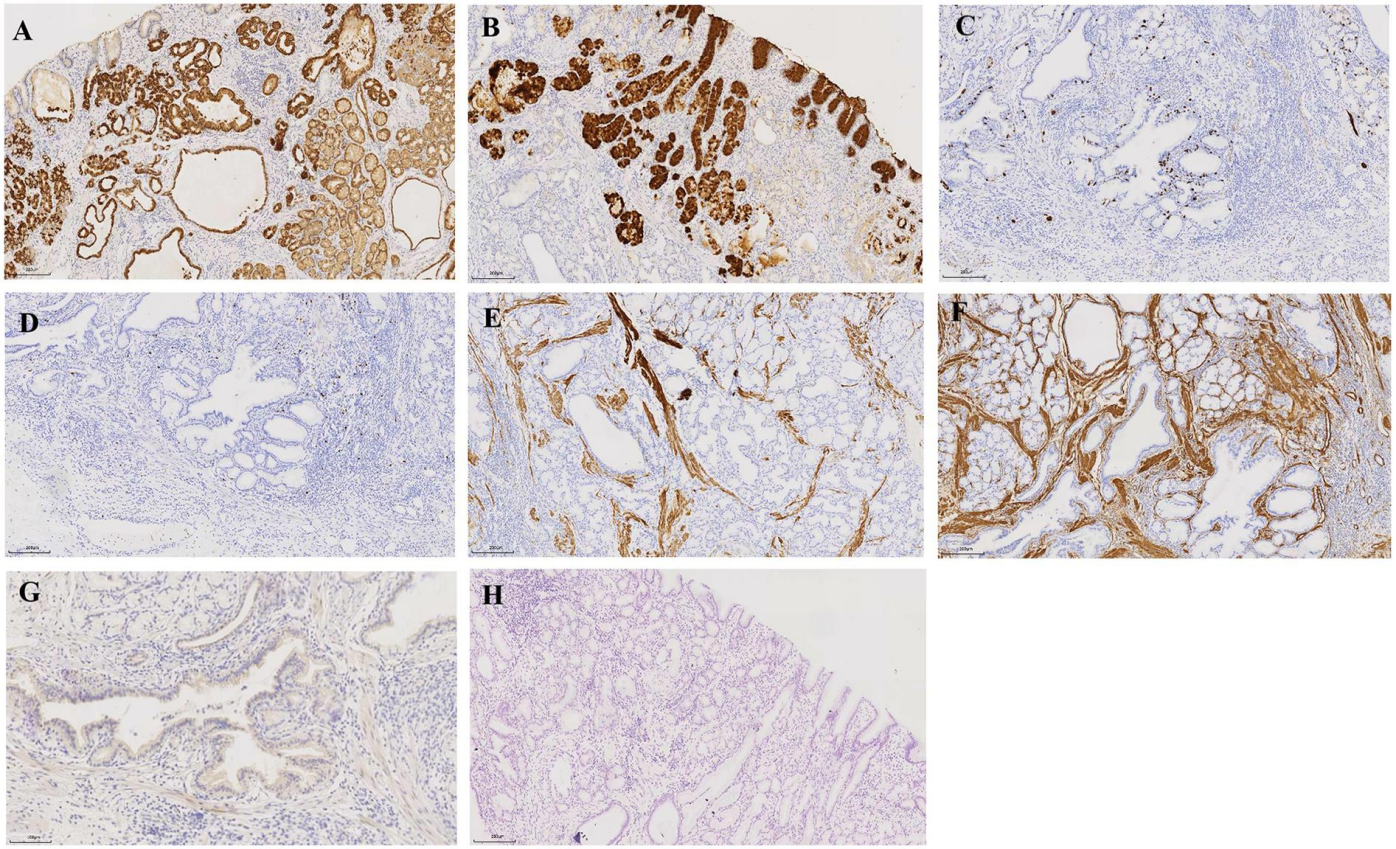
Pathology findings. Immunohistochemistry: **(A)** MUC5AC (+), **(B)** MUC6 (+), **(C)** Syn (+), **(D)** Ki-67 (+, approximately 5%), **(E)** Desmin (+), **(F)** SMA (+), as well as **(G)** MUC2 (−). HE staining: Microscopy of the endoscopic resection specimen revealed hypertrophy of the polyp component in the submucosal layer **(H)** Bar, 200 μm.

Histological examination of the tumor revealed a proliferation of the mucosal layer characterized by glandular and cystic structures, which exhibited no cytological atypia. The surface of the polyp was lined with gastric mucosa resembling either fundic or pyloric gland types. The glandular formations comprised a variety of epithelial cell types, including pyloric or mucous-neck cells, surface mucous (foveolar) cells, and parietal-like cells. Immunohistochemistry results showed: MUC5AC (+), MUC6 (+), Syn (+), Ki-67 (+, approximately 5%), Desmin (+), SMA (+), as well as MUC2 (−) (Figure 3). Based on the endoscopic findings and histopathological features, the diagnosis of an inverted polyp associated with a cardia hamartoma was established. The patient was discharged 7 days post-ESD without any complications.

## Clinical timeline and follow up

**Figure 4:**
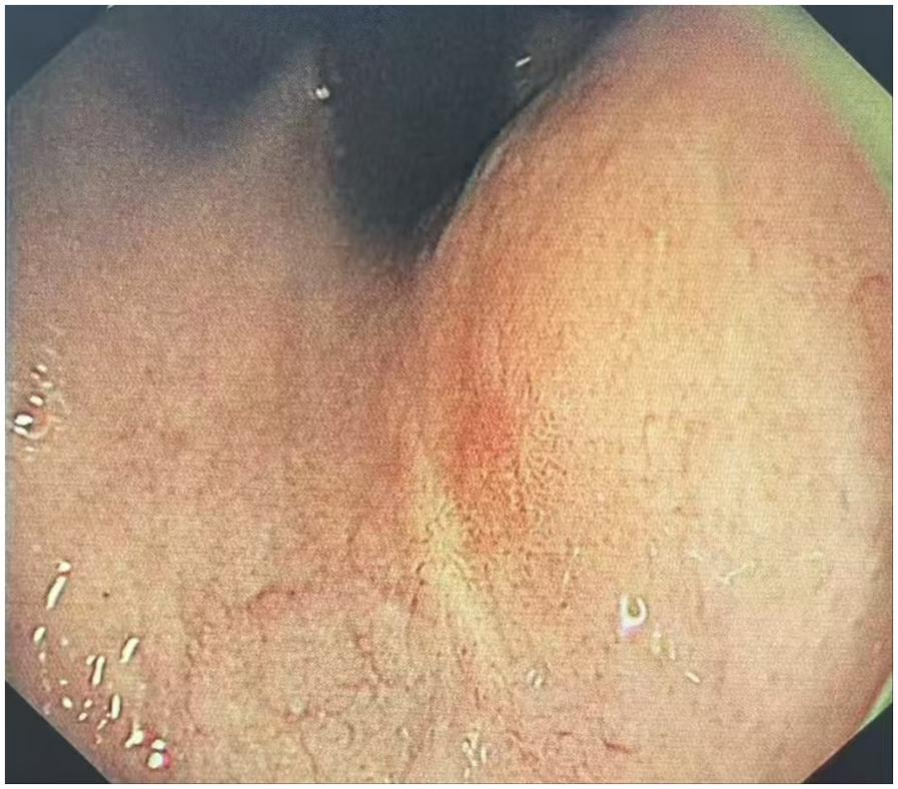
Endoscopic performance at 6 months post-ESD. The results indicated that the patient's cardia mucosa was smooth and without protrusions.

The clinical timeline was as follows: *T* = 0 (initial presentation); *T* = 2 days (ESD procedure); *T* = 7 days (discharge). A follow-up visit was conducted at *T* = 6 months, during which the patient underwent gastroscopy in the outpatient department (Table 1). The results indicated that the patient's cardia mucosa was smooth and without protrusions (see Figure 4). Given the documented, albeit rare, association between GHIP and the development of gastric adenocarcinoma (3), a long-term monitoring plan was established. The patient is scheduled for repeat annual endoscopic surveillance for a minimum of 5 years. Furthermore, due to the known correlation with chronic mucosal inflammation, the patient's status for H. pylori infection and atrophic gastritis will be monitored closely; if present, more frequent biannual endoscopy would be recommended (4).

**Table 1:** Clinical timeline of diagnosis and management for this case.

Time point	Event	Key findings/Actions
*T* = 0 (Admission)	Initial presentation	74-year-old male with a 3-year history of abdominal bloating. No familial cancer history.
*T* = 0	Diagnostic endoscopy & EUS	White light endoscopy revealed a smooth SMT (∼1.0 cm) in the cardia. EUS showed a heterogeneous mass originating from the submucosal layer (Figures 1A,B).
*T* = +2 days	Therapeutic procedure: ESD	Successful *en bloc* resection of the lesion using a Flush Knife 2.0BT. The tumor base was connected to the muscularis propria (Figures 2A–F).
*T* = +7 days	Discharge	Patient discharged in stable condition, having experienced no post-procedural complications (e.g., bleeding, perforation).
*T* = +6 months	First follow-up	Surveillance gastroscopy showed a well-healed resection site with smooth cardia mucosa and no recurrence (Figure 4).

ESD, endoscopic submucosal dissection; EUS, endoscopic ultrasonography.

## Brief patient perspective

The patient provided perspective on his experience, stating: The constant bloating that bothered me for years is finally gone after the procedure. I feel relieved to have a clear diagnosis and am happy with the result. This highlights the significant improvement in quality of life following the successful endoscopic treatment.

## Discussion

Gastric lesions associated with familial colonic polyposis, referred to as GHIP, constitute less than 1% of all gastric polyps (5). Typically, GHIP is asymptomatic; however, some patients may experience abdominal discomfort without distinct clinical features. In certain instances, it may manifest as intestinal obstruction or anemia resulting from chronic blood loss (6, 7). Additionally, the condition has been linked to deep cystic gastritis (3). In the case presented, the patient exhibited no specific clinical symptoms, aside from abdominal bloating. Previous studies indicate that there is no significant gender bias in the occurrence of these lesions, which are considered to be acquired rather than congenital, as they predominantly arise in older adults (2).

Gastric hamartomatous polyps are classified as benign lesions from a pathological perspective; however, approximately 20% of these polyps are found to coexist with gastric adenocarcinoma (4). CHIP is a rare lesion (<1% of gastric polyps) with no unique symptoms (e.g., abdominal bloating only). This can lead to misdiagnosis as common gastrointestinal disorders. In some cultural contexts, non-specific symptoms like bloating may be dismissed as “indigestion” or treated with traditional remedies, delaying formal evaluation. Consequently, the diagnosis and management of these polyps present significant clinical challenges. The therapeutic approach for gastric hamartomatous polyps is influenced by the characteristics and dimensions of the tumors. There are two distinct categories of hamartomatous inverted polyps: those that are stalkless, referred to as the “submucosal tumor (SMT) type,” due to their inversion into the submucosal layer, and those that possess a stalk, known as the “polyp type.” Endoscopic resection is typically performed for all stalked polyps, whereas surgical resection is mandated for stalkless polyps (SMT type), as previous studies have indicated that endoscopic resection of these lesions often results in incomplete removal (2). A recent case study identified low-grade intraepithelial neoplasia in certain areas of postoperative pathology, indicating a potential association between gastric hamartomatous inverted polyps (GHIP) and the development of gastric cancer, thereby highlighting the risk of subsequent gastric cancer progression (8). Furthermore, a recent investigation of the adjacent mucosa in ten patients with GHIP revealed that six patients (60%) exhibited atrophic gastritis or gastritis associated with Helicobacter pylori, while four patients (40%) were diagnosed with non-specific gastritis (9).

GHIP is believed to arise from the infiltration of the mucosa through the muscularis layer, as well as from mucosal crevices or defects resulting from recurrent erosion (10). Typically, GHIP is identified as an isolated submucosal mass during endoscopic procedures (11). An endoscopic evaluation conducted by Dohi indicated that the lesion was enveloped by a normal mucous membrane, characterized by a distinct irregular depression at its apex (12). In a case documented by Takuma Okamura, the use of magnification endoscopy and narrow-band imaging revealed a reddened area surrounding the depression, along with an irregular arrangement of villi and pits, lacking a clear demarcation (13). Consequently, diagnosing GHIP based solely on endoscopic findings presents challenges. Endoscopic ultrasonography typically demonstrates heterogeneous tumors with cystic components. In the case reported by Okamura, endoscopic ultrasonography identified a nonuniform tumor with cystic areas situated within the third layer (13). Additionally, the case presented by Moyu Dohi exhibited a heterogeneous tumor originating from the second layer, characterized by small cystic hypoechoic spots observed via ultrasound endoscopy (12). In this instance, endoscopic ultrasonography confirmed the presence of a heterogeneous tumor, predominantly located within the third (submucosal) layer.

Histologically, the tumor is distinguished by submucosal gland hyperplasia and the presence of cystic structures. The polyp's surface exhibits characteristics of either gastric fundus gland type or pyloric gland type gastric mucosa. Furthermore, the glandular architecture comprises various types of epithelial cells, including pyloric or mucous neck cells, as well as surface mucous (foveolar) cells. The submucosal glands or cystic components are linked to the overlying gastric mucosa through defects in the mucosal muscularis (14). However, the pathological findings in previously documented cases typically reveal polycystic structures (15). Recent studies indicate that approximately three-quarters of cases of GHIP exhibit features resembling submucosal tumors, while the endoscopic characteristics, including findings from endoscopic ultrasonography, are often non-specific. Previous study indicated GHIP coexisted with gastric adenocarcinoma (4). Case reports documented adenocarcinoma arising within GHIPs (3, 13). Low-grade neoplasia has been identified in GHIP resection specimens (8). Potential mechanisms include chronic mucosal injury from inverted gland proliferation, inflammation-driven carcinogenesis (60% of GHIP patients have H. pylori/atrophy) (9), cystic dilation and glandular obstruction, et al. Consequently, achieving an endoscopic diagnosis of GHIP may pose challenges, necessitating complete endoscopic resection for definitive pathological evaluation (16). In the case presented herein, histological examination revealed that the GHIP was characterized by a singular cystic structure located in the submucosa, which was not connected to the surface mucosa. The cyst's surface was lined with foveolar cells and mucinous neck cells.

SMT-type CHIP (stalkless, >1 cm) requires ESD for complete resection, while incomplete resection risks recurrence or missed malignancy. Meanwhile, ESD may be unavailable in rural/underfunded hospitals, forcing referral delays. Previous studies have advocated for surgical resection as the preferred approach for the complete removal of gastric hamartomatous polyps of the SMT type. In the case presented herein, the submucosal tumor in the stomach measured greater than 1.0 cm in diameter. Traditional EMR techniques, however, necessitate advanced skills and have proven reliable primarily for lesions measuring 10 mm or less, as they can be resected in a single piece (17). Consequently, achieving complete resection of larger tumors via conventional EMR methods is deemed challenging. The advent of a novel technique employing an insulation-tipped diathermic knife has demonstrated that performing circumferential mucosal incisions around lesions prior to snaring can enhance *en bloc* resection rates, thereby broadening the applicability of EMR (18). Numerous reports indicate that for lesions measuring 10 mm or greater, the ESD method is preferred to avoid piecemeal resection, which may yield insufficient tissue for accurate pathological evaluation (19). Moreover, endoscopic resection offers several advantages over traditional surgical methods, including reduced invasiveness and cost-effectiveness (20). Notably, recent research has highlighted the efficacy of ESD for gastric hamartomatous inverted polyps, utilizing a clip with a line attachment prior to incision (21).

Repeat endoscopy at 3–6 months post-ESD was need to assess resection site healing. Annual endoscopy for ≥5 years should be conducted, given reports of adenocarcinoma developing during follow-up (13). Those with H. pylori infection or atrophic gastritis require biannual endoscopy (4). For this case, a follow-up visit was conducted for the patient (*T* = 6-month: follow up), who underwent a gastroscopy in the outpatient department. The results indicated that the patient's cardia mucosa was smooth and without protrusions.

Some single-case limitations should be noted. This case introduced an elderly male patient, while CHIP presentation may differ by age, ethnicity, or geography. No long-term follow-up data provided for this patient. ESD success at a tertiary center may not reflect outcomes in low-resource settings. Single cystic structure in this case instead of typical polycystic GHIPs and the heterogeneity may affect behavior.

Accordingly, we successfully employed the ESD technique to treat the tumor in this case. We recommend that SMT-type hamartomatous inverted polyps larger than 1.0 cm in diameter be managed using the ESD method.

## Data Availability

The original contributions presented in the study are included in the article/Supplementary Material, further inquiries can be directed to the corresponding author.
